# Modifiable factors influencing attention performance in healthy children: insights from a comprehensive school nutrition study

**DOI:** 10.1186/s12889-024-19059-8

**Published:** 2024-06-19

**Authors:** Peggy Ober, Tanja Poulain, Christof Meigen, Ulrike Spielau, Carolin Sobek, Wieland Kiess, Ulrike Igel, Tobias Lipek, Mandy Vogel

**Affiliations:** 1 LIFE Child - Leipzig Research Center for Civilization Diseases, Hospital for Children and Adolescents, Medical Faculty, Leipzig University, 04103 Leipzig, Germany; 2 Center for Pediatric Research (CPL), Hospital for Children and Adolescents, Medical Faculty, Leipzig University, 04103 Leipzig, Germany; 3 Integrated Research and Treatment Center (IFB)Adiposity Diseases, Medical Faculty, Leipzig University, 04103 Leipzig, Germany; 4 Department of Applied Social Science, University of Applied Sciences Erfurt, 99085 Erfurt, Germany

**Keywords:** Attention, Academic performance, Breakfast, Media consumption, School, Sport

## Abstract

**Background:**

There is inconclusive evidence for the effects of various leisure activities on attention performance in children. The literature reports inconsistent associations between activities such as physical activities or media use. To date, no study has thoroughly examined the various factors influencing attentional performance in a larger cohort of healthy children. This study aims to close this research gap.

**Methods:**

From 2018 to 2019, the Leipzig School Nutrition Study collected data from 1215 children and their families. The children report their dietary behavior (using CoCu- Questionnaire), especially their participation in school lunch and their breakfast habits, through a paper questionnaire. Furthermore, attention performance was assessed using a validated test (FAIR-2) at school. Data on physical activity, media consumption, family eating habits and socio-economic status (SES) were collected from parents using questionnaires. Associations between attention and influencing factors were estimated using hierarchical linear regression. Analyses were adjusted for age, SES, and school type.

**Results:**

Attending upper secondary schools (ß_adj_= 23.6, *p* < 0.001) and having a higher SES (ß= 1.28, *p* < 0.001) was associated with higher attention performance. Children doing leisure-time sports (ß_adj_= 4.18, *p* = 0.046) or reading books for at least one hour/weekday showed better attention performance (ß_adj_= 3.8, *p* = 0.040). Attention performance was also better in children having no electronic devices in the bedroom (ß_adj_= 13.0, *p* = 0.005) and in children whose parents limited their children’s Internet access (ß_adj_= 5.2, *p* = 0.012). We did not find any association between nutritional habits and attention performance.

**Conclusions:**

We found that fostering modifiable habits such as reading and physical activity could enhance attention performance. These findings have substantial implications for the development of prevention and intervention programs that aim to improve attention in schoolchildren. It is important to note, however, that social status as a hardly modifiable factor also impacts attention performance. Therefore, interventions should address personal habits in a systemic approach considering the child’s social status.

**Trial registration:**

The study is retrospectively registered with the German Clinical Trials Register (DRKS00017317, registration: 05-29-2019).

##  Background

Effective attention performance is essential for children to learn and succeed academically. There is no single definition of attentional performance, but rather various theories and models. Their common thread is the selection of impressions and, if necessary, the prolonged focus on them to further process the gained experiences and knowledge [1].

Attention performance varies with individual developmental stages: Younger children often struggle to maintain attention for extended periods of time, while older children generally have a longer attention span [2]. Attention performance can also be affected by behavioural factors. With the increasing use of electronic media in today’s world, attention is inevitably affected. Studies highlight that excessive screen time and media exposure can negatively impact a child´s attention span and focus [3]. Similarly, Christakis et al. found that early exposure to television is associated with attention problems later in childhood. These studies emphasize the importance of monitoring and limiting children´s media use to promote healthy attention development [4]. However, media consumption encompasses different levels of stimulation and activity. Research indicates that, e.g. video games can also improve attention and academic performance [5, 6].

Compared to the receptive use of electronic media, reading has the potential to enhance attentional performance by training working memory [7]. However, there are only a limited number of controlled studies on the impact of regular reading and being read aloud on attention.

Furthermore, physical activity has been linked to improved attention skills and academic performance in children [8–11]. Potential social and metabolic mechanisms behind this effect including improvement of neuroplasticity are discussed elsewhere [12, 13].

The current literature indicates that healthy nutrition habits in general, and having breakfast in particular, can positively affect attention and school performance [14, 15]. However, the evidence regarding having breakfast is inconclusive. As breakfast is recommended as part of a healthy diet, respective a healthy lifestyle, the associations between breakfast and cognitive function may reflect factors beyond the nutritional aspects [16]. Moreover, not only having breakfast itself but also its quality matters as studies have shown that breakfast of inferior quality had no effect on attention performance [17–19].

Few studies have comprehensively investigated factors influencing attention performance in healthy children. Our study aimed to investigate a wide range of factors potentially influencing attention performance of school-aged children, including nutrition and sports, while adjusting for social status and school environment. Based on previous findings, we expected children´s attention – measured using standardised tests - to be positively associated with regular breakfast and regular participation in sports but negatively with unlimited internet use and low family SES [8, 20]. In addition, the results should provide guidance on the planning and implementation of intervention studies.

##  Methods

###  Study design and sample

Data were collected during the Leipzig School Nutrition Study between May 2018 and May 2019. The Leipzig School Nutrition Study is a cross-sectional study designed to gain a deeper understanding of the relationships between nutrition in the school environment, parent-independent shopping behavior during the school day, and family factors that influence the weight status and attention performance of children in Leipzig [20].

**Fig. 1:**
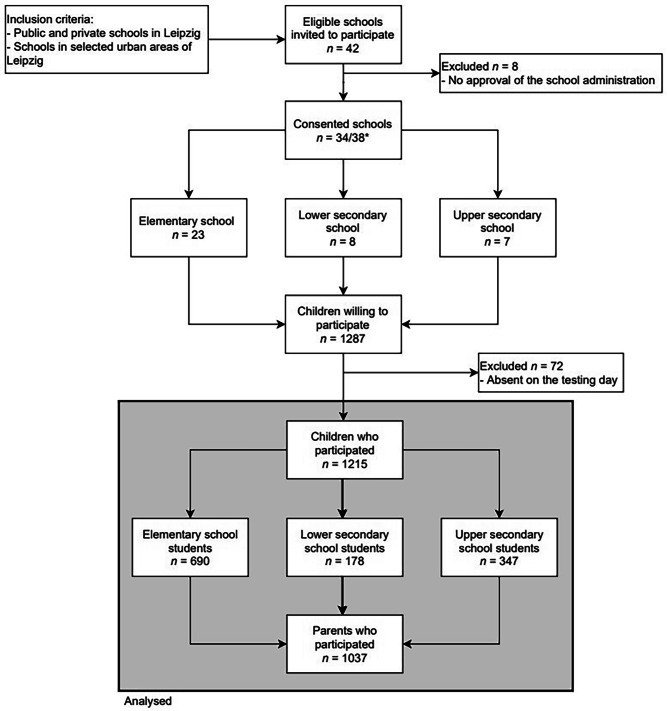
Flow chart visualizing inclusion and exclusion of study participants. * One school comprised an elementary school, a lower secondary school, and an upper secondary school; two schools comprised an elementary school and an upper secondary school. Within the schools comprising more than one type, each class and, therefore, each student, could be assigned one type, the information ultimately used in regression modeling

Forty-two schools in the city of Leipzig from different study areas, differing in their social and structural conditions, were eligible for participation [21]. Thirty-four of these resident schools participated (Fig. 1). After approval of the school administration, all classes of the 4th level (elementary schools equivalent to ISCED1 (According to the UNESCO International Standard Classification of Education [22]) and the 6th − 8th level (secondary schools lower resp. upper secondary schools equivalent to ISCED2 resp. ISCED3) were requested to participate. 1215 children and parents followed this invitation (rates are shown in Fig. 1). Written informed consent was given by the student’s parents. Whereas the participating children received an incentive of 5 euros, the schools did not receive any financial benefits. The study was designed in accordance with the Declaration of Helsinki. The Ethical Committee at the Medical Faculty of Leipzig University (number 483/17-ek) approved the study. The Ethical Committee is registered as an Institutional Review Board with the Office for Human Research Protection (IORG0001320 and IRB00001750). Further, the study is registered within the German Clinical Trials Register (DRKS00017317,registration: 05-29-2019).

### Setting

The survey took place during school hours in the schools before lunchtime, where the study team set up a sufficiently large examination room (primarily a classroom). Only the children with valid consent were present. Parents completed a paper- pencil questionnaire at home.

###  Instruments

The “Frankfurter Aufmerksamkeits-Inventar 2 (FAIR-2)” was used to examine attention performance [23]. It is a validated paper-pencil test suitable for a group setting and appropriate for an age of ≥ 9 years. The test leader instructed the children. Immediately afterward, the two parts of the test were performed, each taking three minutes without a break. Testing was latest completed by 11:30 a.m. The test and its internal and external validity are described in detail elsewhere [24]. All children participated in the attention test. The marker value M was determined according to the scoring protocol to ascertain instructional understanding. Only the tests of children reaching a sufficient M score were included in the analyses. FAIR-2 results in scores of selective attention (FAIR-L = achievement value), continuity of attention (FAIR-K = continuity value), and cognitive self-control (FAIR-Q = Quality value) [25], which were converted into age-adjusted percentiles. Except for the analysis of the correlation between attention and age (use of the unadjusted raw values of the K-score), all analyses are based on these age-adjusted K-score. These percentiles were taken from two age-specific norm Tables (9–13 and 14–17 years). Because few children (*n* = 23) were over 14 years of age and their scores differed considerably from those of the younger group, they were excluded from the analysis.

Children’s questionnaire:

The children’s dietary behavior was assessed using a paper-pencil questionnaire. For the present analysis, we asked the children whether they eat in the school canteen (yes/no). Breakfast habits were assessed by asking if they usually eat breakfast on school days. Response options were “No” / “Yes, at home before I go to school” / “Yes, at school – I buy something” / “Yes, at school – I bring something from home”. Multiple answers were allowed.

Parent’s questionnaire:

The parent’s questionnaire included a short questionnaire for assessing the composition of the child’s diet and culture of eating (“CoCu - Composition and Culture of Eating”) [26]. For the assessment of diet composition, we calculated the “Nutritional Health Score” (NHS) as described in Poulain et al. The score ranges from − 120 to + 120, with higher values indicating a healthier diet [8]. The following yes-no questions were asked in order to ascertain the culture of eating. Does your child usually eat dinner together with the family? Is the TV usually running at home during dinner, or is a tablet, smartphone, cell phone, or similar being used? Does your child usually snack between meals (e.g., chocolate, gummy bears, potato chips, pretzel sticks)? Does your child help you to prepare meals?

The parent’s questionnaire also included items on leisure time behavior, such as the child’s sports activities. For better comparability of the data and later research questions, we have adopted some questions from the study questionnaire of the LIFE Child study (Published e.g. in: [8, 27]). We ask How often does your child spend time (at least 1 h at a time) outdoors? Participants had to choose one of five response categories ranging from “never” to “> Almost every day”. Which of the following sports activities does your child participate in during his/her leisure time? “Do sports as a member of a sports club” / “Do sports outside of a sports club (except school sports lessons)” / “None of the listed sporting activities”. The last question from the sport category relates to the previous question: How often does your child do these activities? “Less often than 1 time per week” / “1–2 times per week” / “3–5 times per week” / “Almost every day” for every previous answer.

Questions assessing media consumption considered the usage of TV, game console, PC with and without internet, adapted from Poulain et al. [28]. Additionally, we inquired about How much time does your child spent reading printed books and magazines on a normal weekday (alone or with a caregiver)? the parents could choose “>4 h per day” / “approximately 3–4 hours per day” / “approximately 1–2 hours per day” / “approximately 30 min per day”/ “not at all”. Finally, 4 yes-no questions were asked about media consumption. Is your child´s Internet use in his or her leisure time (at home and/ or mobile) limited in time by you or other caregivers? Does your child have a TV in his/her room permanently? Does your child permanently have a PC/laptop/tablet in his or her room? Does your child have access to the internet in his/her leisure time (at home and/or mobile)?

The socioeconomic index (SES) was calculated according to the modified Winkler Index [29], a composite score combining information on the parent’s highest level of education, current occupational position, and equivalised disposable income. The score can vary from 3 to 21 and was categorized into low (< 8.5), moderate (8.5–15.4), and high (> 15.4) SES, based on the KiGGS study (German Health Interview and Examination Survey for children and adolescents) [30]. A migration background was assumed if at least one parent was born abroad.

###  Statistical analysis

Descriptive statistics were reported as mean and standard deviation for continuous variables and count and percentage for categorical variables.

Associations between the continuous outcome attentional performance (FAIR-K-score) and independent variables were estimated using hierarchical linear models comprising regression, analysis of variance and analysis of covariance, adjusting for age, sex, and school type if necessary. The effects are presented as coefficients (β) resulting from the models. For continuous independent variables, β corresponds to a slope and describes, therefore, the change in the dependent variable for a one-unit increase of the independent variable. For categorical variables, β corresponds to the difference in the dependent variable compared to the reference group. School type (categorical: primary, lower and upper secondary school), SES (continuous score), migration background (dichotomized yes/no), breakfast on schooldays (dichotomized breakfast/no breakfast), school canteen (yes/no), NHS (continuous score), eating habits (yes/no), sports(dichotomized sports activity/no sports activity), time outdoors (dichotomized into more than two times or less), limiting internet use (yes/no), and presence of electronic media in the child’s bedroom (yes/no) were included as independent variables. For the analysis, we consider all sports equally regardless of whether they are performed inside or outside of a sports club, except for school sports lessons, which were not considered. To account for cluster effects within schools, the school was added as random intercept to the models.

In the cases of high multicollinearity resulting in variance inflation, we removed the respective model terms. This was especially true for models containing both school type and age because primary school only comprised Grade 4 and the secondary schools only comprised Grades 6 and 7 resulting in a high dependency of age and school type. Therefore, models containing the school type were not adjusted for age. We decomposed the individual SES into school means and the individual deviation [31]. The model showed that the individual deviation was no longer significant in our models. Thus, we adopted the mean school SES for all children in the individual school. Interactions between the independent variables and the covariates were tested.

Study data were collected and managed using REDCap electronic data capture tools [32]. All analyses were conducted using R version 4.0 [33]. The level of significance was set to α = 0.05.

##  Results

###  Characteristic of the study sample

Thirty-four schools were included in the study (Fig. 1). A total of 3107 children in Grades 4 and 6–8 were eligible for inclusion in the study by being a pupil of a participating school. The participation rate of children in one school varied considerably between school types. In elementary schools, 43.6% (*n* = 690), in lower secondary schools, 26.9% (*n* = 178), and in upper secondary schools 47.2% (*n* = 347) of children participated. In total, 1215 children and 1037 (85%) parents participated. The children (49.2% male, 50.8% female) were 9 to 15 years old. Basic characteristics of the study population are shown in Table 1.

**Table 1:** Description of the study sample

Study Population (*n* = 1215)			
	Mean (SD)	Range	*n* (%)
Age (years)	11.3 (1.4)	8.9–15.4	
Sex			
Male			598 (49.2)
Female			617 (50.8)
School type			
Elementary school			690 (56.8)
Age (years)	10.3 (0.6)	9.11–12.6	
Lower secondary school			178 (14.7)
Age (years)	12.9 (0.8)	11.0-15.4	
Upper secondary school			347 (28.6)
Age (years)	12.5 (0.8)	8.9–15.3	
SES group			
Low			90 (10.7)
Medium			467 (55.3)
High			287 (34.0)
Missing			371
Migration background			
Yes			196 (19.6)
No			804 (80.4)
Missing			215

Descriptives are given as means, standard deviations and ranges for continuous variables and counts and percentages for categorical variables

###  Attention performance and associations with age and sex

**Fig. 2:**
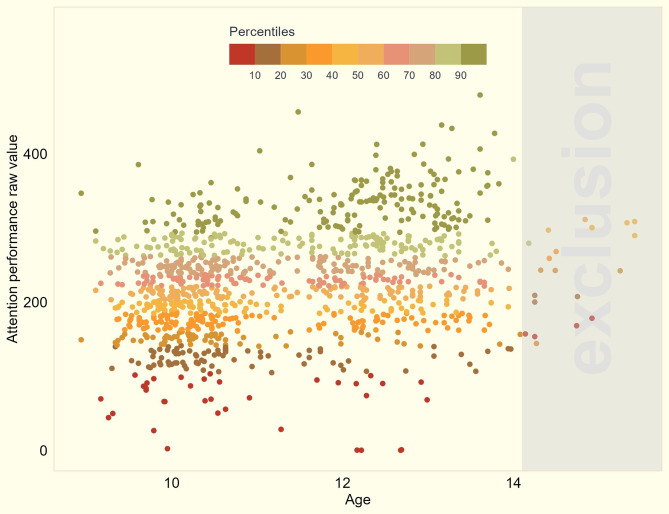
Distribution of raw values of the attention test (K-values) by age. Coloring is performed according to percentile ranks for age

All children (*n* = 1215) participated in the attention test, of which 1121 (92%) reached a sufficient M score. Figure 2 shows the raw K-values of the individual tests.

Raw attention performance was strongly dependent on age across the whole sample, increasing by 16.3 points for each year older (95%CI = 13.3–19.2, *p* < 0.001).

*N* = 1098 tests were available for the age group < 14, and therefore, were included in further analyses. These children achieved a mean percentile score of 60.6 for attention performance. In contrast to age, attention performance was independent of sex, girls achieved on average merely 1.9 performance points less than boys (95%CI= -10.4-6.5, *p* = 0.655). An overview of the regression results is shown in Table 2.

**Table 2:** Results of the linear models assessing the associations between the covariates and attention performance

Variables	Univariate				Multivariate		
	β	95%CI	*p-*value		β	95%CI	*p-*value
*SES*	1.3	0.7–1.8	**< 0.001**		-		
*Age*	16.3**	13.3–19.2	**< 0.001**		-		
*Sex (Ref: Male)*							
Female	1.9**	-10.4–6.5	0.655		-		
*Migration background (Ref: No)*							
Yes	-2.3	-7.3–2.8	0.385		-		
*School type (Ref: Elementary school)*							
Lower secondary school	8.5	1.2–18.8	**0.022**		-		
Upper secondary school	23.6	18.2–29.1	**< 0.001**		-		
**Media Consumption**							
*Limitation online time (Ref: No)*							
Yes	4.3	0.3–8.4	**0.035**		5.2‡†	1.1–9.2	**0.012**
*Stratum school type*							
* Elementary school (Ref: No)*							
Yes	7.6	1.5–13.7	**0.015**		7.0‡	0.9–13.1	**0.024**
* Secondary schools (Ref: No)*							
Yes	5.2	-0.1–10.4	0.051		4.5‡	-0.7–9.7	0.093
*Internet access (Ref: No)*							
Yes	1.4	-3.8–6.6	0.602		1.5‡	-3.7–6.7	0.561
*Own media device*							
* Grade 4 (Ref: No)*							
Yes	-0.1	-5.4–5.2	0.971		0.5‡	-4.8–5.7	0.858
* Grade 6 (Ref: No)*							
Yes	-4.6	-10.5–1.2	0.121		-4.6‡	-10.4–1.3	0.124
* Grade 7 (Ref: No)*							
Yes	-12.7	-21.8 - -3.6	**0.006**		-13.0‡	-22.0 - -3.9	**0.005**
*Reading books*							
* Weekdays (Ref: No)*							
Yes	4.4	0.7–8.1	**0.019**		3.8‡†	0.2–7.4	**0.040**
* Weekend (Ref: No)*							
Yes	3.6	0.3–6.9	**0.032**		3.5‡†	0.2–6.8	**0.038**
**Leisure-time**							
*Leisure-time sports (Ref: Yes)*							
No leisure-time sports	-4.5	-8.6 - -0.3	**0.034**		-4.2‡	-8.3 - -0.1	**0.046**
*Time outdoors (Ref:< 2 times per week)*							
3–5 times per week or >	-2.9	-6.5–0.9	0.112		-3.0‡	-6.6–0.6	0.098
**Nutrition**							
*Breakfast (Ref: Yes)*							
No	3.3	-2.4–9.0	0.257		3.4‡*	-2.3–9.0	0.242
Number of breakfasts (Ref: 0)							
1	-5.9	-12.7–0.9	0.088		-6.4‡*	-13.1–0.3	0.060
2	-6.1	-12.9–0.7	0.080		-6.2‡*	-12.9–0.5	0.070
*Snacks (Ref: No)*							
Yes	-1.3	-4.6–2.1	0.464		-1.57‡†	-4.9–1.8	0.359
*Child helps with cooking (Ref: No)*							
Yes	-1.8	-4.7–1.1	0.217		-1.9‡†	-4.7–0.9	0.202
*Mobile phone/TV usage during dinner (Ref: Yes)*							
No	1.8	-2.3–5.8	0.398		1.3‡†	-2.8–5.3	0.531
*School lunch participation (Ref: No)*							
Yes	0.0	-3.6–3.7	0.981		-0.9‡	-4.5–2.8	0.650
*NHS*	0.6	0.0–1.2	**0.049**		0.7†	0.1–1.3	**0.023**
					0.2‡†	-0.4–0.7	0.618

For each covariate of attention performance, the unadjusted (univariable regression model) and the adjusted effects (multivariable regression model) are reported. Adjustment was initially done for school type, age, sex, and socio-economic status. Unnecessary predictor variables were removed by stepwise backward deletion until only significant covariates remained in the model, following the principle of parsimony. The variables remaining in the model are indicated as follows: *School type †Age ‡SES. Sex turned out to be non-significant in all models. In the univariate analysis, the effect of age and sex was estimated using performance points rather than percentiles because of the age-adjusting transformation into percentiles**. Further, age dependency was estimated across the whole age span, and not restricted to just under 14-year-olds.

###  School type and demographic factors

**Fig. 3:**
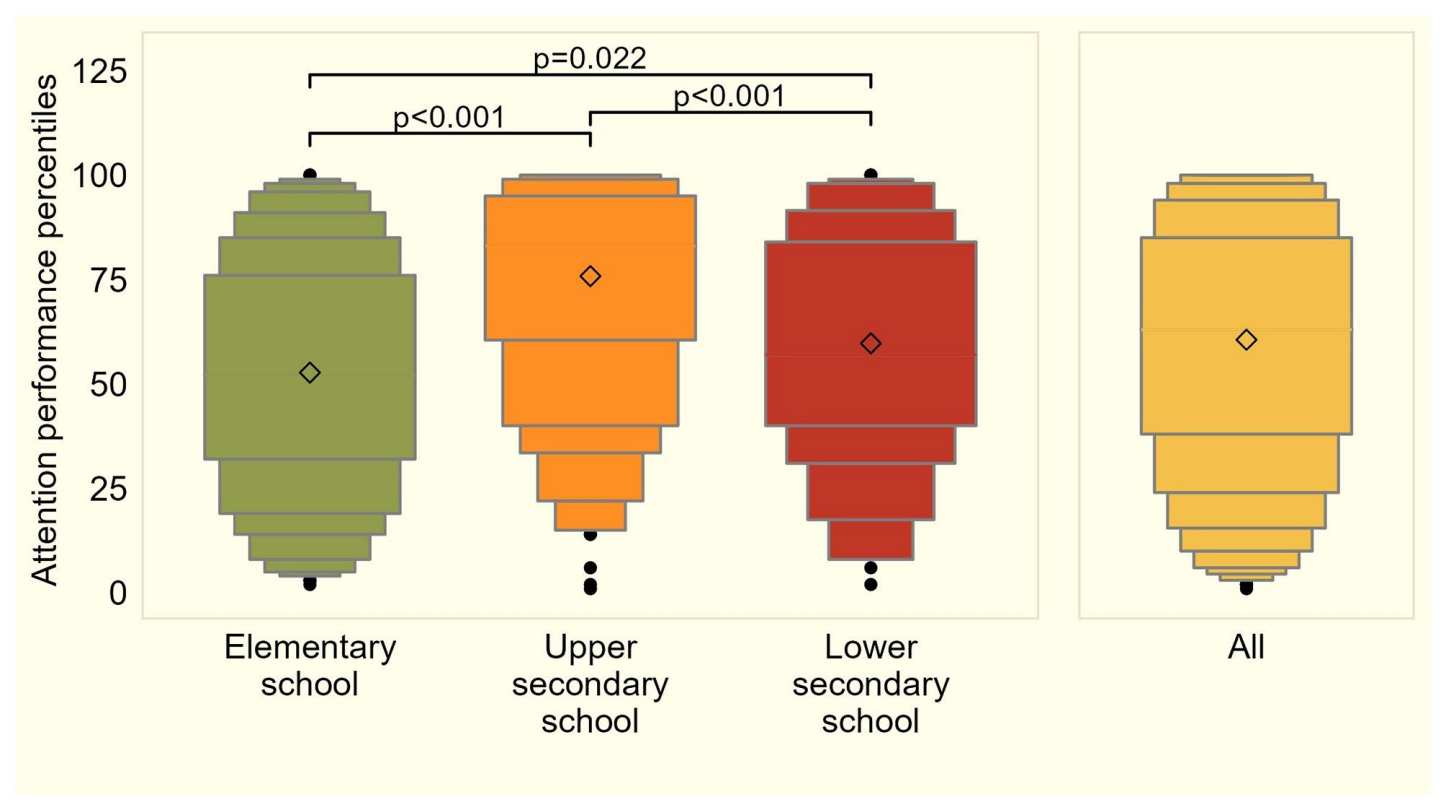
Boxplots showing the distributions of attention performance percentiles by school type

As shown in Fig. 3, elementary school children reached a mean attention performance percentile of 51.8. Compared to these children, children at upper secondary schools reached 23.6 higher attention performance percentiles (95% CI = 18.2–29.1, *p* < 0.001) whereas children of lower secondary schools reached only 8.5 higher attention performance percentiles than primary school children (95%CI = 1.2–15.8, *p* = 0.022).

Attention performance increased with increasing social status. An increase of 1 in a student´s family´s SES, was associated with an increase of 1.3 percentiles in attention performance (95%CI = 0.7–1.8, *p* < 0.001). This association was independent of sex. Children with migration background had an attention performance 2.3 percentiles lower than children without migration background (95%CI= -7.3–2.8, *p* = 0.385).

###  Media consumption

Parental limitation of online time decreased with increasing child age. In Grade 4, internet access was limited in 86% of the cases. In secondary schools, this proportion dropped significantly to 70% and 59% in Grades 6 and 7, respectively. Children whose parents limit their internet use reached a 5.2 higher attention performance percentile than children whose parents did not limit their Internet access (95%CI = 1.1–9.2, *p* = 0.012). The effect was more substantial among elementary school children (ß_adj_= 7.0, 95%CI = 0.9–13.1, *p* = 0.024). For children in secondary schools, the effect size was significantly smaller and lost its significance (ß_adj_= 4.5, 95%CI= -0.7–9.7, *p* = 0.093). The general access to the Internet in leisure time was not related to the attention performance.

**Fig. 4:**
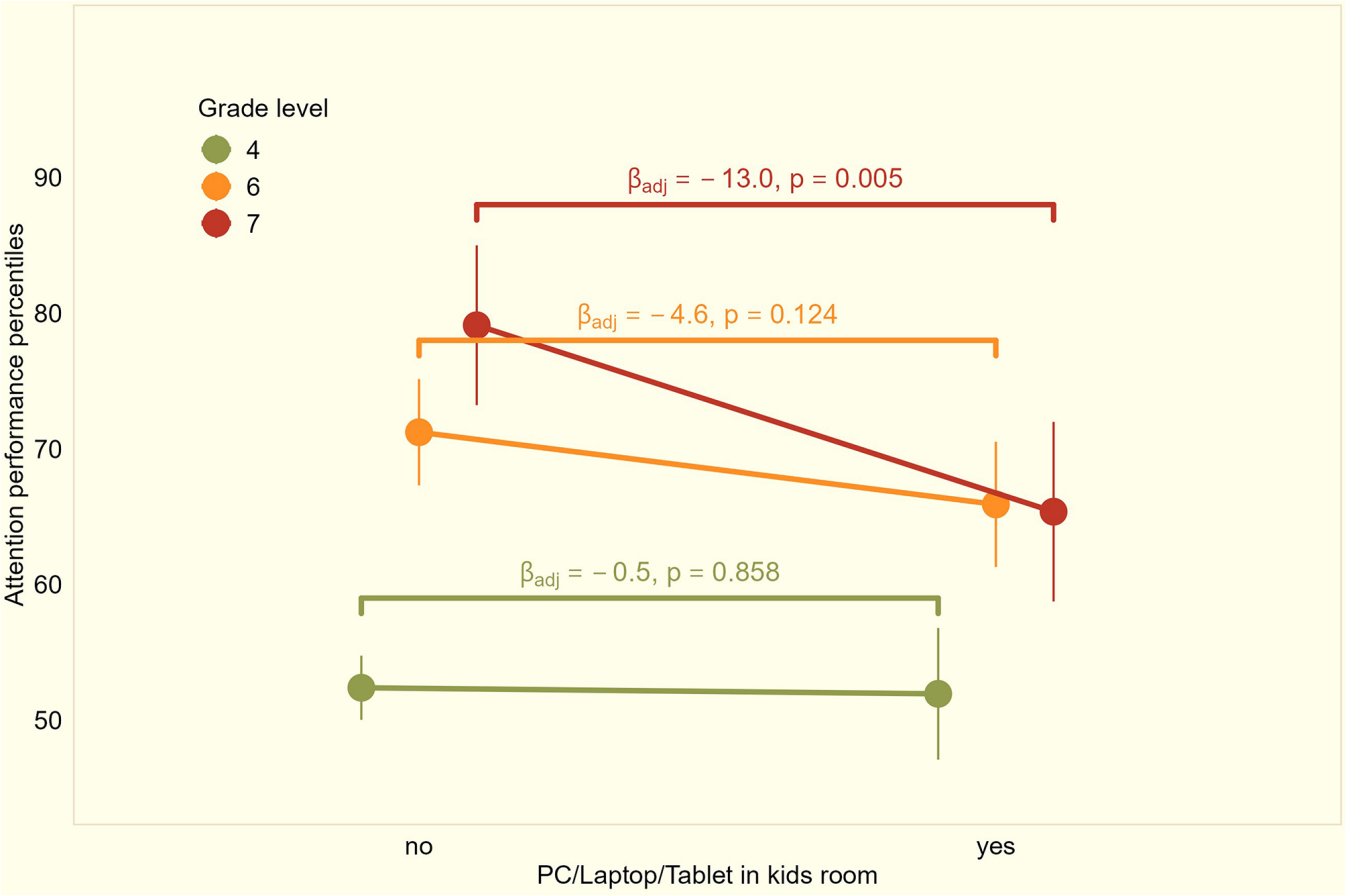
Distribution of attention performance percentiles by presence of an electronic device such as PC/laptop/tablet in the child´s bedroom stratified by Grade (Grade 4 *n* = 552; Grade 6 *n* = 279; Grade 7 *n* = 115)

19% of the children in Grade 4 reported having at least one media device, such as a PC / laptop and/or tablet, in their room. The number doubled between Grades 4 and 6/7 to 42%. In Grades 4 and 6, we observed no statistically significant association between the presence of a media device in the room and attention performance (Fig. 4). However, in Grade 7, children who had at least one electronic media device, in their room reached an attention performance 13 percentiles lower than children without media device in the bedroom (ß_adj_= -13.0, 95%CI= -22.0 - -3.9, *p* = 0.005).

Children who read for at least one hour per day during the week showed a 4 percentiles higher attention performance. The effect persisted after adjustment (ß_adj_= 3.8, 95%CI = 0.2–7.4, *p* = 0.040). There was a similar effect of reading on weekends (ß_adj_.=3.5, 95%CI = 0.2–6.8, *p* = 0.038). The effect of reading on e-book readers could not be evaluated because of low usage rates.

###  Sports and time outdoors

In total, 915 (83%) children did sports regularly in their free time, while 183 (17%) did no leisure-time sports at all. Children doing no leisure-time sports reached a 4.5 lower attention performance percentile than those who engaged in leisure-time sports (ß= -4.5, 95%CI= -8.6 - -0.3, *p* = 0.034). Notably, after correcting for social status, the effect size was similar and remained statistically significant (ß_adj_= -4.2, 95%CI= -8.3 - -0.1, *p* = 0.046).

Only a few parents indicated that their child spends time outdoors less than once a week (*n* = 48, 4%). Children spending 3–5 times per week or almost every day time outdoors (*n* = 659, 60%) showed poorer attention performance than children who spent less time outdoors. However, the effect did not reach statistical significance.

###  Nutrition

A total of 1013 (92%) children regularly ate breakfast on school days (85 children did not eat breakfast). Overall, children who did not eat breakfast on school days showed a slightly, non-significantly better attention performance (ß_adj_= 3.4, 95%CI= -2.3–9.0, *p* = 0.242). There was no consistent effect across the different school types.

Also, we observed no significant association between attention performance and the number of breakfasts eaten (0,1,2). Further, there was no significant association between attention performance and culture of eating like mobile phone/TV usage during dinner or whether the child helps with cooking. Due to the low number of children having no family dinner, its association with attention performance could not be evaluated.

Children who had a higher NHS showed better attention performance: For each increase of 1 in NHS they reached a 0.7 percentiles higher attention performance (95%CI = 0.1–1.3, *p* = 0.023 adjusted for age). However, the effect lost its significance after adjusting additionally for SES (ß_adj_= 0.2, 95%CI= -0.4–0.7, *p* = 0.618), suggesting a considerable correlation between SES and NHS. There was no significant association between school lunch participation and attentional performance (ß_adj_= -0.9, 95%CI= -4.5–2.8, *p* = 0.650).

##  Discussion

###  Main results

In our study conducted with 1215 German schoolchildren, we aimed to investigate a wide range of factors that affect the attentional performance of children. Unlike many other studies, attention performance was evaluated using standardized tests within the school environment. The objective was to identify possible intervention options to improve the children’s attention. We found that children who engaged in sports and read books during their leisure time had better attentional performance. Furthermore, the study also showed that children who did not have electronic devices in their bedrooms and those whose parents limited their online time also had better attentional performance. In addition, we found the non-modifiable factors of SES and school type were strongly related to attention performance. Thus, the hypothesis that the family’s social status influences the child´s attention performance could be confirmed. However, the study was unable to identify any significant associations between nutrition, such as diet quality, regular breakfast meals or participation in school lunches, and attentional performance.

###  Attention performance and leisure time behavior

*Media Consumption*.

Our results show that limiting online time is associated with higher attention across school types, and even after adjusting for social status, the effect remained. However, the effect was stronger for elementary school children. The weaker effect for secondary school children lost its significance after adjustment for SES. This may be due to differing group sizes: Younger children’s online time was more likely to be limited by their parents. Further, results from the KIM-study [34] suggest that younger children’s parents pay more attention to the online content their children consume and are more likely to accompany them while being online. The older the children are, the more frequently they are online - and the more and more unaccompanied. Limiting online access might be linked with better sleep [35] and more physical activity [36] as children have fewer opportunities to spend their time with online activities. Further, less online time often means less time in social networks, an online activity often associated with poorer concentration [37]. The effect is independent of social status [34]. However, we only had dichotomous information and no detailed information on the duration of online time.

Interestingly, whether or not there was internet access was not related to attention performance. We did not collect information on frequency, duration or type of internet use, but considering these characteristics might be crucial. In the KIM-study, 40% of children aged six to thirteen reported being online daily or almost daily, and another 41% accessed the internet once/several times a week [34].

The representative KIM-study showed that German households were fully equipped with TV, Internet access, and cell phones/smartphones. Among the participants, one-third had their children’s room furnished with a TV and 19% with a computer or laptop [34]. Our data are comparable and show that the percentage of children with a PC in their room doubles from grade 4 to 6 but does not increase much from grade 6 to 7. The association between electronic devices in children’s rooms and decreased attention performance becomes significant in grade 7. This could be explained by the onset of puberty and less parental control in connection with developing negative health behavior [38] like excessive use of social media. Again, sleep shortages [39] and the negative consequences of social media usage [37] may impair attention abilities.

Reading books for at least one hour a day was positively related to attention, which aligns with several studies [7, 34, 40]. Reading and even having something read to them increases a child’s vocabulary [7], improves the (working) memory [7, 40], relaxes, and reduces stress [41–43]. Studies also show that children with attention problems have poorer reading performance [44, 45]. Indeed, in general, the direction of the causation cannot be determined, which is also true for our data.

*Sports*.

Children who did not participate in sports achieved poorer attention performance. This finding is in line with previous studies, where physical activity was positively associated with cognition and academic performance [9, 46]. Hillman et al. found that “fitness was positively associated with neuroelectric indices of attention and working memory and response speed in children” [47, p. 1967]. They see fitness as an important promoter of cognitive function and cognitive health in children [46, 47].

We pooled organized and non-organized sports because we wanted to include all activities perceived as sports by the participants. Engaging in leisurely, non-organized sports, like playing basketball in the park, may enhance social skills more than physical skills, technique and endurance. Nevertheless, because it was not possible to gather detailed information on which sport activity at which intensity was regardless of club membership, it was difficult to rate the actual physical activity level. That could be one reason why we found no further associations between the frequency or the duration of sports and attention.

###  Attention performance and Nutrition

We found no statistically significant association between nutrition parameters and attention performance. According to the current literature, better attention performance would be expected for children who eat breakfast regularly [14, 15]. However, the quality of the breakfast is also essential, as negative correlations were found for a low-quality breakfast [17, 18]. In our study, we did not ask about breakfast composition; only frequency and place were recorded. Furthermore, the time interval between breakfast and the attention test was variable as the tests started at different times. However, the test was always completed before noon to reduce potential influence of daytime, as is recommended by the manual.

In general, questionnaires are not ideal for capturing nutrition [48]. On the one hand, people find it challenging to estimate portions; on the other hand, it is difficult to remember and estimate what and how much one (or one’s children) has eaten on average across and after a certain period. The CoCu offers a convenient opportunity to assess basic characteristics of nutritional behavior [26]. However, the association between the NHS as a basic measure of diet quality and attention lost its significance after adjustment for SES, which most likely reflects the close link between health-related behavior and social traits.

As expected, we did not find an association between having lunch and attention since the attention tests were always completed before lunch. School lunch and cognition studies are rare and have shown no effects on attention [49]. However, a regularly consumed warm lunch can be considered as a proxy for a healthier diet in general, which has been shown to impact various learning outcomes positively [50].

###  Attention performance and SES

In line with previous studies, we found a strong positive association between social status and attention performance [51, 52], which is also true for the relationship between SES and intelligence and academic performance. Both correlates strongly with attention and later educational and occupational outcomes [53]. Children from low social status backgrounds have limited developmental opportunities compared to their better-off peers, especially in Germany [54] as most of extracurricular activities are subject to fees. Therefore, it is widely agreed that socially disadvantaged children should receive exceptional support to compensate for their disadvantage [53–55].

We found that the type of school was also significantly associated with attention performance - independent of social status. In contrast to the family’s social status, where the direction of the effect is clear, the relation between school type and attention might be ambiguous or bi-directional. Do children in a lower secondary school have poorer attention performance, or do they not have access to a higher type of school because of low attention performance?

###  Opportunities for intervention

School type and social status significantly impact attention and school success. The literature shows neither the family’s social status nor school type are promising intervention targets. Even if intervention measures were easily accessible or tailor-made for families with precarious backgrounds, most interventions that aimed at individuals or families failed to succeed [56]. Therefore, systemic approaches seem to be more promising. On the other hand, we found several associations between health-related behavior and attention performance. Engaging in such activities requires in most cases parental or institutional support, whether financial or educational. Children lacking such support from their parents have to rely on kindergartens and schools. However, in Germany, these institutions are overstrained to make the appropriate offers (e.g. sports, cooking). There are initial efforts toward mitigating social disparities. Every family with low income can apply for the “education and participation package” for their children. The package covers fees for sports clubs, music lessons, lunch, tutoring, and school materials at least partly [57].

Nevertheless, the school environment offers opportunities for interventions to reach children independent of their families’ social status and parental involvement. An example is setting up early morning sports for school classes supplementary to physical education classes [58]. A German study showed that sports interventions (45 min of sports at school three times a week or jogging sessions) could improve social behavior and attention [59]. Indeed, promoting joint intra- and extracurricular activities like school sports, but also other activities, may help reduce social disparities and, therefore, foster academic success and a sense of community. Another example is a comprehensive media education ingrained in the general and subject-specific curricula. There are already effective time-limited programs to prevent problematic media use and addiction. It might be beneficial to integrate these programs into the curriculum [59–62] as school-based interventions are predestined to equally reach children with high and low SES respectively [63].

###  Strengths and weaknesses

The study data were based on questionnaires, which were completed by parents and children, and, therefore, prone to social desirability bias. As already discussed, it is difficult to capture nutrition using questionnaires. Further, the use of questionnaires in general is usually afflicted by reporting and recall biases. Nonetheless, direct measurement or observation was not feasible in our study.

We measured attention performance directly with a validated test. The test is approved for a group setting and thus measures attention directly in the school. The test conditions were standardized, and the children were shielded from external influences as far as possible. The age classification of the reference tables led to unfavorable group sizes (n aged ≤ 14 = 1098 and n aged > 14 = 23). We had to exclude the children from the older age group, as their percentiles differed considerably. This difference was obviously related to age, which would have caused significant distortions in the analyses.

The Leipzig School Nutrition Study analyzed data from numerous children and parents on nutrition, leisure, and purchasing behavior. The schools were all located in the urban area of Leipzig, the eighth-largest city in Germany [64]. As is also generally the case in studies, the social status was somewhat higher than in the general population [65]. By design, the districts were selected based on socioeconomic features and the prevalence of overweight, aiming for a well-balanced population [21]. The study’s high participation rate in the schools ensured high representativeness.

##  Conclusions

The study aimed to identify modifiable factors that may serve as possible intervention targets, thereby enhancing children’s attention. Our results suggest that besides the non-modifiable factors school type and social status, extracurricular sports activities and daily reading may improve attentional performance. Furthermore, we could show that parental time limits on Internet access were associated with better attention performance. The former two can be addressed within the school and the family context, while the latter depends on parental compliance. Therefore, low-threshold access to sports activities and the encouragement of daily reading should be considered in subsequent school-based interventions.

## Data Availability

The legal requirements and the given informed consent do not allow public sharing of the dataset. Interested researchers can contact the research data management of the Medical Faculty, University Leipzig: forschungsdaten@medizin.uni-leipzig.de for further information. The dataset ID is PID-00080/01.

## Abbreviations

CoCu: Composition and Culture of Eating Questionnaire
FAIR-2: Frankfurter Aufmerksamkeits-Inventar 2
ISCED: International Standard Classification of Education
KiGGS: Cohort longitudinal study on the health of children, adolescents and young adults in Germany
KIM: Series of studies (“Children, Internet, Media”)
NHS: Nutritional Health Score
PC: personal computer
PISA: Programme for International Student Assessment
SES: Socio-economic status
